# Eye-Tracker Study of the Developmental Eye Movement Test in Young People without Binocular Dysfunctions

**DOI:** 10.3390/life13030773

**Published:** 2023-03-13

**Authors:** Elvira Orduna-Hospital, Aitana Navarro-Marqués, Carmen López-de-la-Fuente, Ana Sanchez-Cano

**Affiliations:** Department of Applied Physics, University of Zaragoza, 50009 Zaragoza, Spain

**Keywords:** DEM, developmental eye movement test, eye movement conjugacy, eye-tracker, fixations, ocular motility, saccades

## Abstract

Background: The purpose of this study was to evaluate ocular motility in normal young adults when performing the Developmental Eye Movement (DEM) test using an infrared eye-tracker in a sample of young subjects without visual dysfunctions. Methods: An optometric evaluation was carried out on 52 participants with a mean age of 21.00 ± 3.22 years to verify they did not have any binocular dysfunction, by completing a computerized version of the DEM test while their eye movements were recorded with an eye-tracker. A custom-written software was developed to analyse some specific parameters of ocular motility while performing each subtest (Test A, Test B and Test C) of the complete DEM test. Results: The mean duration of the fixations was shorter in Test C (243.56 ± 46.18 s) than in Test A (493.52 ± 171.41 s) and Test B (484.20 ± 156.59 s). The mean adjusted horizontal (AdjHT: 35.24 ± 6.68 s) and vertical (VT: 33.58 ± 5.56 s) times were at the 45th and at the 40th percentile, respectively. In Test C, there was a high positive significant correlation between the saccadic speed (cc: 0.77; *p* < 0.001) and the saccadic length (cc: 0.74; *p* < 0.001) of both eyes. Conclusions: The eye-tracker is an objective method to evaluate the DEM test in subjects without binocular dysfunctions, measuring and quantifying ocular motility parameters that are impossible with the traditional subjective method. The eye movements of both eyes are conjugated in each subject, having saccades of the same length and speed.

## 1. Introduction

The vast majority of activities require very high visual performance. Most of the perceived information enters through sight, which is one of the main sensory capacities to learn and relate to our environment. A visual function that we must master from an early age is reading ability since reading difficulties have a significant impact on both academic and work success [1]. In this activity, eye movements must be space–time coordinated, otherwise double vision will ensue. They play a very important role since, when reading saccades, fixations and backwards movements or regressions are performed, which require both eyes to move and work conjugately [2,3,4].

The Developmental Eye Movement (DEM) Test is a timed test controlled by the examiner, which measures visual–verbal ability by evaluating eye movements, specifically saccadic movements, through three subtests. This test is related to automatic naming of numbers, visual processing speed and reading ability, which can help identify people at risk of reading problems [5,6,7,8,9]. In addition, poor execution of this test could imply a risk in academic learning since oculomotor skills and visual attention are not adequate or not yet fully developed [6]. Currently, technology allows the recording of ocular motility both objectively and accurately. Eye-trackers are electronic devices that use invisible near-infrared light and high-definition cameras to project light into the subject’s eye, resulting in corneal reflections, which are recorded. Advanced algorithms are then used to calculate eye position and the direction of gaze, previously calibrated for each subject. This makes it possible to measure and study visual behaviour and eye movements in each position, as well as the position of each eye and how they are relative to each other. It provides unbiased, objective and quantifiable data and offers information in real time; with live transmission, it shows the gaze direction of the person immediately [10]. With this device, it is possible to objectify eye movements and relate them to reading ability [11] or, as other authors have done, while performing the DEM Test [5,6,12,13], but almost all of them in children, we used the eye-tracker to collect data during the DEM in young, normal adults (18–30 years of age). Such data are not available since most of the pertinent studies have been performed in children.

Using eye tracking to record both vertical and horizontal eye movements is useful in exploring the underlying mechanisms of eye movement behaviour while reading multiple lines of text organized in paragraphs; since fixating upon words in these multiline arrangements requires accurate ocular motility in both the vertical and horizontal directions [5]. Therefore, the main objective of this work is to objectively measure with an eye-tracker the ocular motility parameters that intervene when performing the DEM Test and to evaluate the coordination between both eyes in a sample of young subjects without visual dysfunctions. With this novel study, it is intended to calculate the normative percentiles and the quantitative values of fixations and saccades while the test is performed in this population.

## 2. Materials and Methods

### 2.1. Sample Description

This study included 52 healthy subjects from 18 to 30 years of age. It was approved by the Comité de Ética de Investigación de la Comunidad de Aragón (CEICA) with reference PI21-074, and the conduct of the study adhered to the tenets of the Declaration of Helsinki. Written assent was also obtained from all of them on the day of examination.

Optometric tests prior to the DEM Test were carried out to ensure that the participants did not suffer from binocular vision dysfunctions or ocular pathologies. In addition, they had to meet the following inclusion criteria: having best corrected visual acuity equal to or greater than 0.8 (20/25 on the Snellen chart) in both eyes, refractive error between −6.00 D and +3.00 D of spherical equivalent, with less than 1.50 D of astigmatism, people without systemic or ocular pathologies and not having used electronic devices one hour before the measurements.

### 2.2. Optometric Exam

With the optometric examination, the functionality of the visual system was evaluated under optimal lighting conditions in each subject and always by the same examiner between 10:00 a.m. and 1:00 p.m.

The procedure was as follows: best corrected visual acuity measurement, cover test, the Northeastern State University College of Optometry (NSUCO) oculomotor test to assess subjective pursuits, saccades and fixations, near point of convergence, the Worth test to avoid suppressions, stereopsis measurement, positive and negative fusional vergence measurement both in near and far vision and accommodative (+/−2.00 D) and vergence (3∆ base-in/12∆ base-out) facility in near vision.

### 2.3. Experimental Protocol

**Figure 1:**
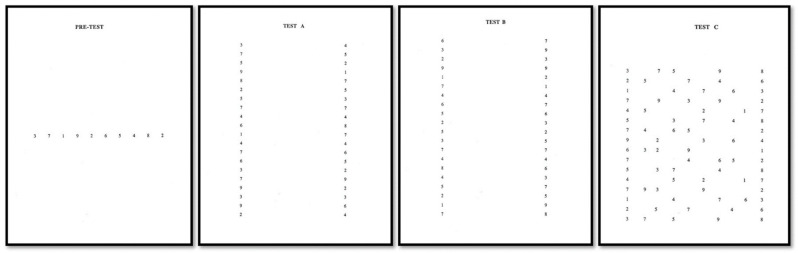
The four calibrated slides of the Developmental Eye Movement Test. Pre-test, Test A, Test B and Test C.

Once any problem of vergence or accommodation had been ruled out, ocular motility was evaluated using the DEM Test monitored with an eye-tracker. Before performing the three diagnostic DEM Test sheets, the Pre-test was performed (Figure 1), where knowledge of numbers was evaluated. If errors were made in the Pre-test, it was not recommended to use the DEM Test in that person. The first two sheets were Test A and Test B, which involve a vertical reading of 40 numbers each, placed in two columns of 20 numbers each (Figure 1). These two tests served to detect problems at the motor level or visual–verbal recognition of numbers since they require automaticity to recognize them. Finally, the third test (Test C) corresponded to a horizontal reading of 80 numbers arranged in 16 rows (Figure 1). In this Test C, peripheral vision intervenes to accurately initiate and terminate the saccade and its fixation field amplitude [14].

The complete test consists of the subject reading all the numbers of Test A, Test B and Test C as quickly as possible and aloud without pause but controlling and trying not to make mistakes or skip any (as it is a purely visual ability, using a finger to guide the reading is not allowed). Meanwhile, the examiner recorded the last, in seconds, for each vertical and horizontal sheet and the errors made, whether they were deletion, substitution or addition of a number, by the subject in a specific template.

For each participant the vertical time (VT) was calculated by adding the times of Tests A and B [6]. To calculate the adjusted horizontal time (AdjHT), Equation (1) was used, and the ratio was found by dividing the AdjHT by the VT (Equation (2)):

AdjHT = [HT × 80/(80 − o + a)](1)

(o, omissions; a, additions)

Ratio = (AdjHT/VT)(2)

The eye tracking device used was the Tobii Pro Fusion eye-tracker (Tobii AB, Danderyd, Sweden), with a dual-camera system and two pupil tracking modes (bright and dark pupil), with dimensions of 374 × 18 × 13.7 mm and capturing gaze data at speeds of 250 Hz. It was connected to a computer where the Tobii Pro Fusion eye-tracker programs were installed: the eye-tracker Manager (Tobii AB, Danderyd, Sweden) for screen selection and the Tobii Pro Lab (Tobii AB, Danderyd, Sweden) for calibration in each examination and where the recordings and their subsequent segmentation were made.

**Figure 2:**
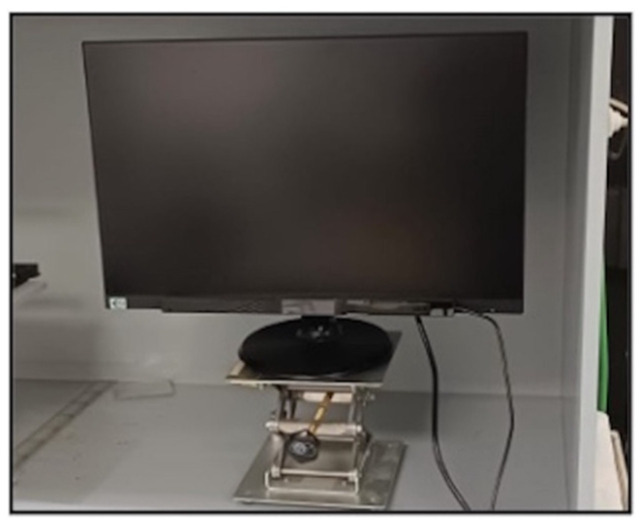
Assembly of the screen and the eye-tracker in the lighting box.

The experimental set-up consisted of a 23-inch screen located inside a cabinet with neutral grey-coloured walls and illuminated with cool white LEDs (6670K correlated colour temperature) that were used to achieve a controlled lighting level over the screen; thus, 945.65 lx reached the monitor surface (Figure 2).

The DEM Test was digitized and calibrated for this screen size to control its projection during the experiments.

The participants were seated with their chin and forehead resting on the chin rest 60 cm from the screen with the DEM Test calibrated for a visual acuity of 0.8. The eye-tracker was placed just below the screen at 60 cm from the participants.

After explaining to the participants how to perform the 4 subtests of the DEM Test (Pre-test, Test A, Test B and Test C) (Figure 1), we calibrated the eye-tracker with a nine-point calibration, including calibration points at each of the four corners of the screen, and asked the participant to read out loud the number for the record and for monitoring the performance of the 4 subtests in one session. Thereby, we were able to recognize errors and omissions it took to complete the test. It should be noted that while the test was carried out objectively with the eye-tracker, the examiner timed subjectively each subtest and noted in a template if they made any errors to exclude those who made an error since a sample without oculomotor problems was required.

### 2.4. Data Collection

**Figure 3:**
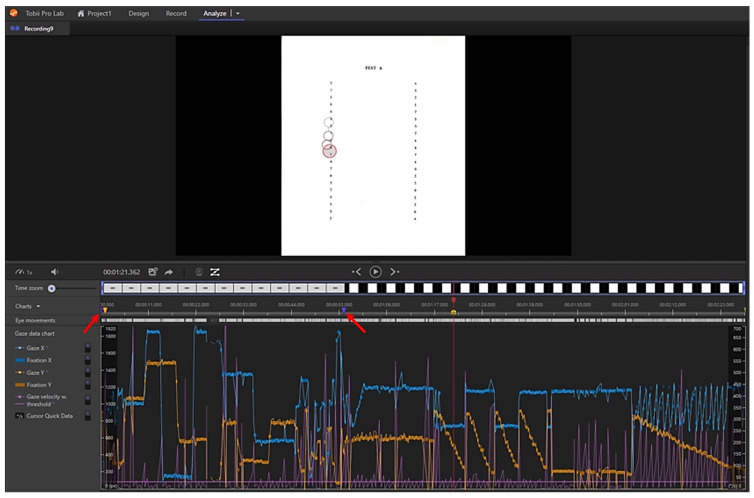
A complete recording made to a participant with the Tobii Pro Lab program. Above the Test A with the grey-red circles indicating where the subject is looking at that moment. Just below is the complete recording with the possibility of reproduction for its segmentation into events, the first part corresponding to the calibration, continuing with the Pre-test and Tests A, B and C. Next, an event marked with two purple triangles (indicated by two red arrows) belonging to the start and end of the Pre-Test event. Below, the graphic representation of the gaze tracking, fixations and saccades.

All recordings were reviewed and segmented with the Tobii Pro Lab program. In it, the selected time intervals (“events”), between the two triangles indicated in Figure 3, were established. These events corresponded to the first and last fixations made by the subjects in each subtest; thus, each recording was segmented into four parts that were analysed separately and the data from each recording individually (one per subject) were exported to Excel (Microsoft Office Excel 2011, Microsoft Corporation, Redmond, WA, USA).

**Figure 4:**
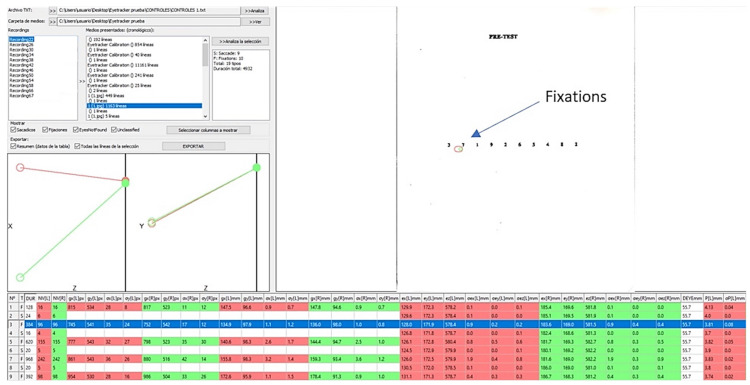
Analysis of the Pre-test “event” with the Etracker Parse program. In the boxes on the left are the recordings to be analysed, each of them segmented into four events. Just below, the people’s eyes are represented in the horizontal (x) and vertical (y) planes and where their visual axes meet. On the left is a Pre-Test image with two fixations, the green one belonging to the right eye (RE) and the red one to the left eye (LE) (indicated with a blue arrow). In the grid below with red (RE) and green (LE) backgrounds, the values of the variables of interest to be analysed. The line marked in blue corresponds to the two fixations (RE and LE) represented in the Pre-Test image.

A custom-made in-house program called Etracker Parse (University of Zaragoza, Zaragoza, Spain) was created to analyze the parameters of interest. With this program (Figure 4), the following variables could be determined for each subtest separately for the same subject thanks to the “events” established in the first program: each subtest duration (s), number (n) and mean duration (s) of saccades and fixations, the interpupillary distance at each moment (mm), right eye (RE) and left eye (LE) pupil size (mm), length (mm) and speed (m/s) of the RE and LE saccades separately and mean saccadic and fixation duration (s).

These data were re-exported to Excel and grouped into three much more manageable databases divided by subtest (Test A, Test B and Test C), with the variables of all the recordings together for the statistical analysis. In addition, the times measured subjectively by the examiner were added to the database to compare them with those measured by the eye-tracker and to validate the method.

### 2.5. Statistical Analysis

The measurements of the variables to be studied were recorded in three Excel databases. Statistical analysis was performed using the Statistical Package for the Social Sciences (SPSS 20, SPSS Inc., IBM Corporation, Somers, NY, USA). First, descriptive statistics of the sample were performed according to the quantitative variables specified in the previous section for each subtest, calculating the mean, standard deviation, maximum and minimum. Non-normal distribution of the values was assessed with the Kolmogorov–Smirnov test, and Wilcoxon signed-rank test was used to compare both methods for related samples (objectively and subjectively time measurement in each subtest). It was studied whether there were correlations between methods and between the variables for subtest C between eyes of the same subject with the Spearman’s test. A *p*-value < 0.05 was considered statistically significant. Scatter diagrams with regression lines to complete the results were plotted.

## 3. Results

Sixty young, healthy subjects between 18- and 30-years-old were selected. Eight participants were excluded since the eye-tracker had not detected them well and the data were not entirely reliable. The final study was performed with 52 subjects with a mean age of 21.00 ± 3.22 years; 30 were women, and 22 were men. The mean ± standard deviation refractive error was −2.10 ± 2.23 D of spherical equivalent.

Table 1 shows the results of the three subtests measured objectively by the eye-tracker and subjectively by the examiner (duration). There were no significant differences between the times measured with both methods in any subtest (*p* > 0.05). In addition, a significant positive correlation was found in all cases (Test A: cc = 0.701, *p* < 0.001; Test B: cc = 0.827, *p* < 0.001 and Test C: cc = 0.645, *p* < 0.001). Regarding the results obtained from the eye-tracker, the number of saccades was greater than the number of fixations. The mean duration of saccades was longer in Test C (22.90 ± 2.70 ms) than in both Test A (15.94 ± 2.22 ms) and Test B (16.10 ± 1.90 ms), the opposite in the mean duration of the fixations, with shorter duration in Test C (243.56 ± 46.18 ms) than in both Test A (493.52 ± 171.41 ms) and Test B (484.20 ± 156.59 ms). In addition, the time to perform the first two tests was similar (Test A: 16.51 ± 2.83 s vs. Test B: 17.11 ± 2.85 s).

The speed of the saccades was slightly faster with the LE (Test A: 0.95 ± 0.35 m/s, Test B: 0.96 ± 0.53 m/s and Test C: 1.30 ± 0.36 m/s) than with the RE (Test A: 0.85 ± 0.29 m/s, Test B: 0.91 ± 0.36 m/s and Test C: 1.25 ± 0.38 m/s) for all subtests. The saccadic length was also greater with the LE (Test A: 33.45 ± 19.51 mm, Test B: 39.04 ± 25.68 mm and Test C: 46.41 ± 20.46 mm) than with the RE (Test A: 31.43 ± 20.83 mm, Test B: 32.34 ± 22.67 mm and Test C: 38.56 ± 12.41 mm).

The AdjHT, the VT and the ratio (Table 2) were calculated considering the values of all the participants and for both methods, without significant differences between them (*p* > 0.05). A significant positive correlation was also found in all parameters (VT: cc = 0.753, *p* < 0.001; AdjHT: cc = 0.645, *p* < 0.001 and Ratio: cc = 0.715, *p* < 0.001). The AdjHT and the VT did not vary since the subjects who made omissions or additions were excluded so that they did not interfere with the results.

The percentiles for this age group (21.00 ± 3.22 years) were calculated according to the data obtained objectively by the eye-tracker from our 52 subjects (Table 3). For VT and AdjHT, the data were at the 40th and at the 45th percentile, respectively.

For Test C parameters measured with the eye-tracker, it was calculated whether there were correlations between the RE and the LE in terms of the speed and amplitude of the saccades and pupil size (Table 4).

**Figure 5:**
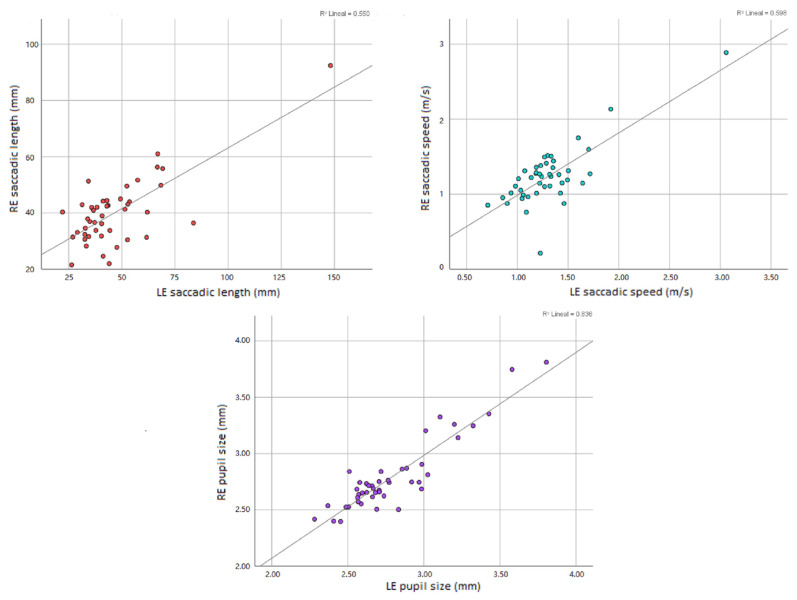
Left: Scatter plot with regression line of the saccadic length of the right eye (RE) by that of the left eye (LE) of each participant for Test C. Right: Scatter diagram with regression line of the saccadic speed of the RE by that of the LE of each participant for Test C. Down: Scatter diagram with regression line of the pupil size of the RE by that of the LE of each participant for Test C.

It was observed that in Test C, there was a high positive significant correlation (cc: 0.77; *p* < 0.001) between the RE saccade speed and the LE saccade speed, as well as with the RE saccade length and that of the LE (cc: 0.74; *p* < 0.001), which means that the greater the speed and length of the RE saccade, the greater the speed and length of the LE saccade in the same person (Figure 5). In turn, we found a statistically significant positive correlation (cc: 0.91; *p* < 0.001) between the RE pupillary size and the LE pupillary size (Table 4 and Figure 5).

## 4. Discussion

The purpose of this study was to objectively measure ocular motility parameters with an eye-tracker when performing the DEM Test, which is a visual–verbal saccadic eye movement task with variable spacing in a sample of young adults without visual dysfunctions. First, it should be noted that no differences were found between the duration of each subtest obtained by the eye-tracker and by the examiner. In addition, there was a highly significant positive correlation between the subtests’ duration measured with both methods. Therefore, the objective method with eye-tracker can be validated.

When obtaining the specific percentiles for our group of subjects, we observed that the mean VT was at the 40th percentile while the mean AdjHT was around the 45th percentile. In the case of the mean ratio, we obtain a 50th percentile; these values could be considered normal since in the DEM Test, normal values are considered from the 31st percentile [14,15].

Comparing our results with those obtained in the DEM Test normative for 13 years [14,15] since it is the age that is closest to that of our subjects, our VT was 33.62 s, slightly lower than the theoretical adjusted VT for 13-year-old subjects, which is 33.75 s. Regarding the AdjHT, the results were similar; our AdjHT was 35.24 s, and the theoretical AdjHT was 37.56 s at 13 years. Regarding the ratio, we obtained a value of 1.05 ± 0.09, a lower value than in the case of the theoretical adjusted ratio for a group of 13 years (1.12 ± 0.12). The subtest times taken by the eye-tracker were more precise since they were considered from the first to the last fixation and without the possible error made when taking it with a chronometer. We obtained lower than expected values for a group of 13-year-old subjects. These results follow the trend that the older the subjects are, the faster the test is performed, as observed in the standardized DEM Test for children in the 6–13 range [14,15].

In this study, we also searched for standardized normal values for the DEM Test in young adults [16,17]. In this case, we looked at the <24 years’ column since the average age of our sample is 21 years. Comparing our obtained times, corresponding to Test A and Test B, with those obtained by Gené-Sampedro [16,17], we observed that our vertical (VT: 33.58 ± 5.56 s) and horizontal (AdjHT: 35.24 ± 6.68 s) times were lower than the values obtained in the DEM Test for adults (Vaj: 52.00 ± 7.0 s and Haj: 55.50 ± 7.5 s). It must be considered that the test designed for adults is two digits, unlike the test designed for children, which is one digit. In the adult test [16], it could be seen that as age advanced, each subtest took longer to complete. Therefore, looking at the results of the DEM Test for children [14,15], ours for 21 years and the DEM Test for adults [16], there was a tendency to reduce the time to perform the test as the age advanced from 6 to 21 years, but from 24 years onwards, it took longer and longer to perform the test.

Comparing the specific data obtained in our study by the eye-tracker, both the speed (Test A: 0.95 ± 0.35 m/s, Test B: 0.96 ± 0.53 m/s, Test C: 1.30 ± 0.36 m/s) and the length (Test A: 33.45 ± 19.51 mm, Test B: 39.04 ± 25.68 mm and Test C: 46.41 ± 20.46 mm) of the saccadic movements, we found that in the horizontal Test C, they were faster than in the vertical Test A and Test B. In addition, we performed more than twice as many saccades in Test C (186.66 ± 96.87) than in Test A or Test B (49.96 ± 41.95 and 53.20 ± 44.46), which may be due to the greater amount of numbers and their spaced horizontal arrangement in Test C. All the subjects were young and did not have any oculomotor dysfunction, so the results obtained were considered within expectations and can serve as a reference for other investigations. Thus, with this study, an attempt was made to obtain percentiles to have a normative table in terms of VT, AdjHT and ratio for the mean age of 21 years, since the traditional DEM Test only has them in the age range from 6 to 13 years [14,18].

In this study, a mean of 189.24 fixations were made, very similar, although slightly less than those found by Hindmarsh et al. [5] in their group of 7.9 ± 0.3 years with average or above reading ability group (199.1 ± 47.31). Moiroud et al. [12] compared eye movements during the DEM Test C between a group of children with dyslexia and a group of children without dyslexia for the same age (9.2 ± 0.4 years), reporting grater fixation counts for the first one (150 vs. 134, respectively). In our group, Test C had a mean fixation count of 121.14, somewhat lower than the people without dyslexia (134) reported by Moiroud et al. [12] but considering that our subjects were older than theirs.

Some studies suggest that visual processing skills may also underline differences in fixation duration [6,13]. In reading eye tracking experiments, fixation duration metrics are used as an indication of online processing speeds and represent the time required to recognize and process the meaning of a word within the text [3,19]. In the present study, fewer fixations were found in Tests A and B (33.90 ± 12.29 and 34.20 ± 9.50, respectively) but of a longer duration (Test A: 493.52 ± 171.41 ms and Test B: 484.20 ± 156.59 ms) while in Test C, there were more fixations (121.14 ± 15.24) but of shorter duration (243.56 ± 46.18 ms). This may be because the numbers in Test C are arranged horizontally, and our eye movements are more trained to read horizontally than vertically. Hindmarsh et al. [5] found that both DEM subtest times and fixation durations were significantly worse in the below-average reading group, which may suggest slower visual processing speeds. This is similar to what Tanke et al. [13] suggested since they found that the duration of fixation during the DEM Test was related to visual processing speed as it was assessed using a timed acuity task. In addition to what was postulated by Ayton et al. [6], performance scores on the horizontal and vertical DEM subtests were associated with visual processing speed when measured by rapid serial visual presentation tests. Therefore, children with slower AdjHT and VT in the DEM Test tended to have slower reading rates [20,21]. Other studies have suggested that the more efficient eye movement patterns displayed by the normal reading group could be attributed to better visuospatial attention skills that facilitate this eye movement behaviour, although more research involving other tests of visuospatial attention, such as spatial cueing [22,23,24] and visual search [25], are necessary to confirm this theory. Inefficient eye movement patterns while reading, including more regressions (right to left saccades), shorter saccadic amplitudes and longer fixation durations have been observed through eye tracking studies in children and adults with both poor reading ability and those diagnosed as having dyslexia compared to good readers [3,19,26,27,28]. In contrast, no differences have yet been found in eye movement patterns when performing everyday tasks unrelated to reading, but between those with good and poor reading ability, it was observed that they have a similar ability to perform eye movements outside the realm of reading [5].

Since the DEM horizontal Test C is a multiline task, children often lose their place while reading along the line, leading to additional vertical eye movements to previous or subsequent lines (different from the expected return sweep movements) [13]. Therefore, we agree with Hindmarsh et al. [5], who propose the analysis of both vertical and horizontal eye movements to understand the behaviors of eye movements during reading. It should be noted that in our sample of young subjects without binocular dysfunctions, it has been observed that the speed and length of the saccadic movements of both eyes have a significant positive correlation, indicating that they work in a coordinated manner, as the pupillary diameter does during the DEM Test (Table 4 and Figure 5). This indicates that evaluating the length and speed of the saccades of each eye of the same subject could help in the diagnosis of people with eye movement coordination problems that affect reading. This is the reason of developing our own software, we wanted to study how each eye works separately and its effectiveness as a whole.

Therefore, there is still controversy about the quantitative results of eye movements and their relationship with reading ability, which shows that further research is needed on the subject to detect both problems in time [6,12,29,30,31]. Thus, they could be treated with visual therapy and corrected so that children can have normal academic progress, or in the case of adults with problems in the ocular musculature due to some illness, medication or accidents, they can be helped to improve them. The DEM Test requires the recognition of numbers presented in a complex spatial matrix. Therefore, the use of eye tracking can allow multiple visual information processing skills to be assessed, such as visual processing speed or spatial attention simultaneously and objectively, especially in cases in which any of these difficulties is suspected since it will be always seen where the people’s visual axes are heading. In the study by Heick et al. [32], they performed the DEM Test and the King–Devick (K–D) Test, noting that they respond equally in concussion assessment, although it appeared to be more efficient to use the DEM Test as part of a multifaceted concussion assessment because it assesses saccadic movements both horizontal and vertical. Gil-Casas et al. [33] showed that subjects with Multiple Sclerosis spent more time than healthy subjects in performing K–D and DEM pseudo-reading tasks, but they can capture impairment in attention, language and other areas that correlate with suboptimal brain function in addition to oculomotor dysfunctions. Thus, visual information processing tests of this nature, monitored with an eye-tracker, can be useful both in research and in clinical settings for a rapid and effective diagnosis and proper therapies. Although further studies are needed in order to evaluate whether the tests used are valid for assessing binocular dysfunction in the general population.

## 5. Conclusions

In conclusion, the eye-tracker probe is an objective device to evaluate the DEM Test in young subjects without binocular dysfunctions, measuring and quantifying ocular motility parameters impossible to evaluate with the traditional subjective method. In addition, it could be considered a complimentary test that is easy to use and quick and accurate for evaluating healthy subjects in the binocular examination. With this novel study, the normative percentiles are calculated and proposed for a group of 21-year-olds without binocular dysfunctions. On the other hand, quantitative values referring to fixations and saccades are obtained while the test is performed, having saccades of the same length and speed between the eyes of the same subject.

## Figures and Tables

**Table 1:** Mean and standard deviation (SD) obtained from the evaluation of the Developmental Eye Movement Test with eye-tracker. Differences between the eye-tracker and the examiner measurements were calculated. A value of *p* < 0.05 was considered statistically significant.

n = 52 Mean ± SD	TEST A			TEST B			TEST C		
	Eye-Tracker	Examiner	(*p*)	Eye-Tracker	Examiner	(*p*)	Eye-Tracker	Examiner	(*p*)
Duration (s)	16.51 ± 2.83	16.62 ± 3.23	0.651	17.11 ± 2.85	17.32 ± 2.83	0.71	35.24 ± 6.70	36.11 ± 2.83	0.532
Number of fixations (n)	33.90 ± 12.29			34.20 ± 9.50			121.14 ± 15.24		
Number of saccades (n)	49.96 ± 41.95			53.20 ± 44.46			186.66 ± 96.87		
Mean duration of saccades (ms)	15.94 ± 2.22			16.10 ± 1.90			22.90 ± 2.70		
Mean duration of fixations (ms)	493.52 ± 171.41			486.20 ± 156.59			243.56 ± 46.18		
Interpupillary distance (mm)	61.52 ± 2.92			61.52 ± 2.92			61.52 ± 2.92		
RE saccadic speed (m/s)	0.85 ± 0.29			0.91 ± 0.36			1.25 ± 0.38		
LE saccadic speed (m/s)	0.95 ± 0.35			0.96 ± 0.53			1.30 ± 0.36		
RE saccadic length (mm)	31.43 ± 20.83			32.34 ± 22.67			38.56 ± 12.41		
LE saccadic length (mm)	33.45 ± 19.51			39.04 ± 25.68			46.41 ± 20.46		

Abbreviations: RE: right eye; LE: left eye.

**Table 2:** Mean and standard deviation of the values of the 52 participants and differences between the eye-tracker and the examiner measurements. A value of *p* < 0.05 was considered statistically significant.

n = 52	VT (s)			AdjHT (s)			Ratio			Errors	
	Eye-Tracker	Examiner	(*p*)	Eye-Tracker	Examiner	(*p*)	Eye-Tracker	Examiner	(*p*)	Eye-Tracker	Examiner
Mean	33.62	33.94	0.683	35.24	36.11	0.534	1.05	1.07	0.723	0.00	0.00
SD (±)	5.56	6.42		6.68	2.83		0.09	0.11		0.00	0.00

Abbreviations: VT, vertical time; AdjHT, adjusted horizontal time, SD, standard deviation.

**Table 3:** Percentiles.

Percentiles	VT (s)	AdjHT (s)	Ratio
1	45.32	49.71	1.21
5	42.87	47.33	1.18
10	41.40	45.48	1.16
15	40.60	42.92	1.14
20	40.22	41.21	1.14
25	38.13	40.06	1.12
30	36.03	38.70	1.10
35	34.95	36.90	1.09
40	33.99	36.18	1.06
45	32.83	35.50	1.06
50	31.13	33.75	1.04
55	30.89	33.42	1.03
60	30.46	32.21	1.03
65	30.41	31.35	1.02
70	30.13	30.42	1.01
75	29.59	29.68	0.99
80	29.08	29.05	0.98
85	28.86	28.32	0.96
90	28.48	27.04	0.92
95	26.28	26.80	0.91
99	24.79	26.03	0.88

Abbreviations: VT, vertical time; AdjHT, adjusted horizontal time.

**Table 4:** Correlation between the right eye (RE) and left eye (LE) of saccadic speed (m/s), saccadic length (mm) and pupil size (mm) for Test C.

Correlation of Related Samples	Saccadic Speed RE/LE (m/s)	Saccadic Length RE/LE (mm)	Pupil Size RE/LE (mm)
TEST C	CC: 0.77 *p* < 0.001	CC: 0.74 *p* < 0.001	CC: 0.91 *p* < 0.001

## Data Availability

Not applicable.
